# GigaDB: promoting data dissemination and reproducibility

**DOI:** 10.1093/database/bau018

**Published:** 2014-03-12

**Authors:** Tam P. Sneddon, Xiao Si Zhe, Scott C. Edmunds, Peter Li, Laurie Goodman, Christopher I. Hunter

**Affiliations:** ^1^Department of Genetics, Stanford University, USA, ^2^GigaScience team, BGI HK Research Institute, 16 Dai Fu Street, Tai Po Industrial Estate, Hong Kong

## Abstract

Often papers are published where the underlying data supporting the research are not made available because of the limitations of making such large data sets publicly and permanently accessible. Even if the raw data are deposited in public archives, the essential analysis intermediaries, scripts or software are frequently not made available, meaning the science is not reproducible. The *GigaScience* journal is attempting to address this issue with the associated data storage and dissemination portal, the *GigaScience* database (GigaDB). Here we present the current version of GigaDB and reveal plans for the next generation of improvements. However, most importantly, we are soliciting responses from you, the users, to ensure that future developments are focused on the data storage and dissemination issues that still need resolving.

**Database URL:** http://www.gigadb.org

## Introduction

The life sciences fields are all embracing the recent advances in technology, whether they are the next-generation sequencing in genetics/genomics (1), high-throughput mass spectrometry in proteomics (2)/metabolomics (3) or the integration of multiple data types in a single analysis (4, 5), with a steady increase in the number of published peer-reviewed articles to match. The accepted ideal of scientific publishing is that researchers provide the data from which they have drawn their conclusions to enable peers and reviewers to retest those data to validate the claims. However, all too often, papers are published where the underlying data are not made available because of factors such as a lack of community repositories or size, meaning there is no opportunity for independent validation of findings. Sometimes the raw data are deposited in archives, but the essential analysis intermediaries, scripts or software are not available, meaning the results of a study are not reproducible by others.

Good science not only finds solutions but also generates more questions. Therefore, the challenge for publishers is not only to provide the resources for scientists to read and validate the results but also to allow them to interrogate the data with their own questions. Despite the majority of high-impact journals setting out editorial policies relating to the public availability and sharing of data, one study showed that <10% of papers deposited full raw data (6).

In addition, with the rapid development of technology comes the *ad hoc* development of many, often untested, software solutions for data analysis. Unfortunately, code availability policies are even more poorly adhered to than data release policies (7). For correct validation of results, the software tools used must also be available and the methods of a paper should include the software versions and parameters used in every step. Some may even argue that hardware can affect analysis, and therefore advocate the creation and use of virtual machines to ensure software always behave consistently. Aiming to incentivize and address this reproducibility gap, here we discuss the current and future state of the *GigaScience* database (GigaDB), covering the rationale and current data publication ecosystem, the relationship and integration with its companion *GigaScience* journal, the types of data held, the use of standards, citation of data by digital object identifier (DOI) and the planned additions and improvements to the database.

## Publishing data

There is a growing awareness of a data reproducibility and access gap, and some funders such as the NSF are starting to mandate data management and sharing plans. There has been a need for infrastructure and mechanisms to facilitate these mandates, as well as calls for better incentives and credit to make the time and effort to do this worthwhile (8). There are a growing number of databases purporting to allow scientists to share their findings in more open and accessible ways, as well as a new generation of data journals trying to leverage them, and it is important to discern where GigaDB fits among these. The ecosystem of biorepositories includes domain-specific databases such as those of the International Nucleotide Sequence Database Consortium (INSDC; GenBank, ENA and DDBJ), as well as the variety of protein and peptide repositories (e.g. PRIDE, AE, GEO), metabolomics (MetaboLights) and imaging repositories (e.g. Morphosource, MorphoBank). There is also now a selection of broad-spectrum databases including GigaDB, Zenodo, FigShare and Dryad. The benefits of the broad-spectrum databases are that they are not restricted by the data types they can hold, meaning researchers can deposit data from the entire set of experiments of a study in a single place. Although these resources cater well for the ‘long-tail’ of data producers working with tabular data in the megabyte to gigabyte size range, researchers working in more data-intensive areas producing high-throughput sequencing, mass-spectrometry and imaging data are not as well served by their file size limitations and charges. GigaDB is able to leverage the tens of petabytes of storage as well as the computational and bioinformatics infrastructure already in place at its host institution, BGI, much cheaper than that of any other publisher, and in a similar manner to Zenodo using the CERN Data Centre.

Although the greater than Moore’s Law growth in the amount of sequencing data produced is well publicized, areas of research such as high-throughput phenotyping and functional genomics screens, brain mapping and super-resolution microscopy are also producing increasingly large volumes of data. Furthermore, with an increasing number of integrative ‘multi-omics’ studies combining genomic, transcriptomic, proteomic and metabolomics data, there is an increasing need for infrastructure to help curate, integrate and present these types of large-scale biological data-oriented studies. The use of cloud-based storage such as Amazon S3, or platforms using this technology, solves many of these issues, but there are cost and stability issues for long-term storage (9), and they are generally not tailored for biological data and metadata.

## Journal: GigaScience

*GigaScience* (10) is an online open-access journal that includes GigaDB as a part of its publishing activities (11). *GigaScience* is co-published in collaboration between the BGI and BioMed Central, the world’s largest genomics organization and first commercial open-access publisher, respectively, to cater for biological and biomedical researchers in the era of ‘big-data’. The journal’s scope covers studies from the entire spectrum of the life sciences that produce and use large-scale data as the center of their work. Studies have shown that citation of work is greatly improved by the availability of associated data (12). Data from *GigaScience* articles are hosted in GigaDB, from where they can be cited to provide a direct link between the study and the data supporting it. The journal also provides a forum for discussions surrounding best practices and issues in handling large-scale data. See http://www.gigasciencejournal.com for additional information about the journal.

## Database: GigaDB

GigaDB primarily serves as a repository to host data and software tools associated with articles in *GigaScience*. It also includes a subset of data sets that is not associated with *GigaScience* articles from the funding organization, BGI, enabling them to release their data quicker, and allowing them to be cited and receive credit for its release before publication in journal articles. With support from China National Genebank, a non-profit institute supported by the government of China and operated by BGI that has database infrastructure as a part of its remit, GigaDB has leveraged and taken advantage of the tens of petabytes of storage and computational infrastructure already in place at the BGI to handle and present large-scale biological data sets much cheaper and easier than other similar resources. A good example of the utility of prepublication release of data was from our first data set, the genome of the deadly 2011 *Escherichia coli* outbreak in Germany (13). Its immediate release into the public domain allowed bioinformaticians around the world to perform rapid, publicly available and openly discussed analyses. This provided crucial data in the fight against the pathogen for 2 months before the ‘open-source genomic’ effort was eventually published in the *New England Journal of Medicine* (14). The rapid release of preliminary data to the research community while enabling their producers to obtain credit through citation is a growing trend with the BGI, and the wider research community, where the speed of data production far outstrips the ability of researchers to analyze and write up their findings.

GigaDB defines a data set as a group of files (e.g. sequencing data, analyses, imaging files, software programs) that are related to and support an article or study. To maximize their utility to the research community, all data sets in GigaDB are placed under a Creative Commons CC0 waiver. This is increasingly accepted as the most appropriate mechanism for dedicating data to the public domain, as it eliminates legal impediments to integration and reuse of large collections of data, such as attribution stacking (15). Although the data are released without legal restriction, scientific etiquette is built on attribution.

## Author attribution and credit

Through association with DataCite (www.datacite.org), each data set in GigaDB is assigned a DOI that can be used as a standard citation for future use of these data in other articles by the authors and other researchers. All data sets in GigaDB require a title that is specific to the data set, an author list and an abstract that provides information specific to the data included within the set. As much metadata as possible is then provided to DataCite to maximize its discoverability in their repository and in the Thomson-Reuters Data Citation Index (http://thomsonreuters.com/data-citation-index/). Following the Digital Curation Centre’s (DCC) best practice formatting and citation guidelines (16), GigaDB data sets are integrated into *GigaScience* through citation in the references, and we have also worked closely with other publishers to ensure that the use of GigaDB data is correctly attributed and cited in their journals as well (17). Citation guidelines are outlined clearly on each data set entry, and functionality to export to reference managers as BibTex files is also included.

In addition to providing mechanisms for citation and reuse, it is important to measure statistics to provide insights into the use of content and further incentivize deposition. DataCite already supplies resolution statistics that are publicly available, and although GigaDB hosts only a small number of data sets at present, the numbers show that GigaDB is receiving over the DataCite average number of hits (http://stats.datacite.org/). GigaDB is keen to help promote and provide alternative metrics for scholarly impact to the journal impact factor, so alternative measures and mechanisms for dissemination and credit are also used, with social media integration and functionality included. The ability to share to Facebook, Twitter and Google+ is provided, and statistics are included at the bottom of every entry. An RSS feed of the latest data sets is also provided.

## Data types

The scope covers not only ‘omics’ type data and the fields of high-throughput biology currently serviced by large public repositories but also the growing range of more difficult-to-access data, such as imaging, neuroscience, ecology, cohort data, systems biology and other new types of large-scale sharable data, as well as software used to analyze large-scale data sets. By archiving software packages and data analyses as executable scripts, for example, all of the pipelines used in the publication of the SOAPdenovo2 genome assembler (18), this incentivizes reproducibility of software papers. By giving DOIs to software, their citeability and longevity is improved while also strengthening the links between publications and code that may not be possible from code repositories.

At the time of writing, GigaDB has issued 64 DOIs to data sets including data from genomic, transcriptomic, metagenomic, epigenetic, proteomic, mass spectrometry, imaging, workflows and software platforms. A total of ∼20 TB of data are currently available to download from the GigaDB servers, with the largest single data set comprising ∼15 TB (19).

It should be noted that GigaDB will only host data that can be released without restriction, and will not host data that are restricted for any reason, be that ethical or otherwise.

## Standards and interoperability

To encourage scientists to submit their data, it is important to make the perceived barrier to data release as low as possible. One way in which GigaDB is doing this already is to provide a multitude of options for submitters, while still ensuring data meets community standards, for example, encourage compliance with the Genome Standard Consortiums (GSC) Minimum Information about any Sequence (MIxS) standard (20). To assist authors in this respect, GigaDB provides a basic spreadsheet template for users to complete and upload in either comma separated value (CSV) or Excel formats. In addition, they are able to accept ISA-Tab (21) formatted files. ISA-Tab is a format used by the BioSharing and ISA Commons communities and is widely used by a growing number of resources and databases such as the Metabolights database (22), Harvard Stem Cell Discovery Engine (23) and Nature’s upcoming *Scientific Data* journal (http://www.nature.com/scientificdata/).

So that these different submission formats do not fragment the data, the information is imported from them into a relational database to be stored in a uniform structure for searching and retrieval. In future releases, tools will be provided to export from the database in a variety of formats via an Application Programming Interface (API) or simple download button on each data set page, thus allowing users to acquire the data set metadata in the format most useful to them.

**Figure 1.**
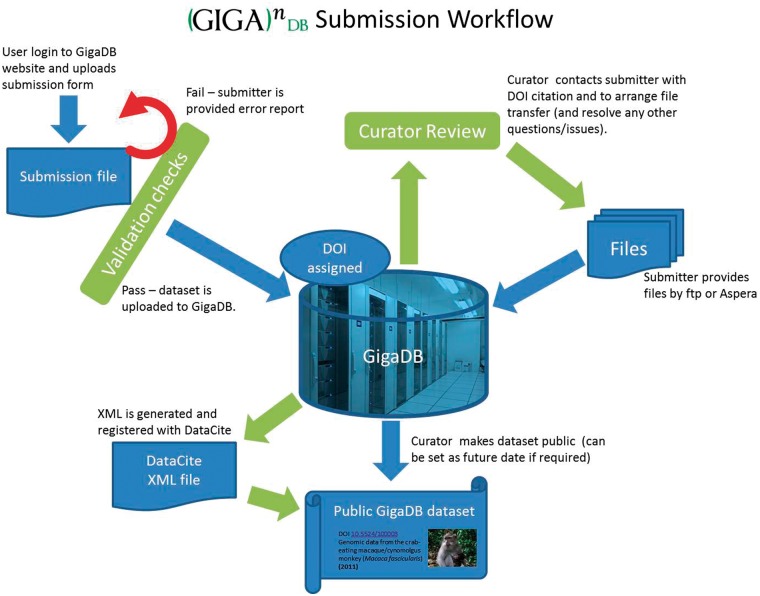
A simplified workflow of data through GigaDB from submission to presentation.

Figure 1 illustrates the submission workflow, with curation of the basic information before and after the archiving of the data. Initially, information is collected and collated about the study and samples, and checked for completeness. This information forms the basis of the DOI, and the data files (sequence, MS/MS, annotations, images, etc.) are then transferred to the GigaDB servers from the submitter. Care is taken to ensure these files are in appropriate formats and are correctly linked to the relevant metadata. Finally, a DOI is minted through DataCite, and release of the data through the GigaDB Web site can occur.

Because of the size of files being moved around, GigaDB makes use of the Aspera software (http://asperasoft.com) to speed up the transfers. In our experience, up to a 30-fold increase in transfer speeds over FTP has been observed, which means that 2 GB of data can be moved in <1 minute instead of 30 minutes on a 1-MBps network connection. A free Web browser plug-in from the developers of Aspera has to be downloaded and installed for users to transfer data to and from GigaDB. However, we are actively looking at alternative open-source methods of high-speed data transfer to keep with the ethos of open data.

## The ‘cyber-centipede’

**Figure 2.**
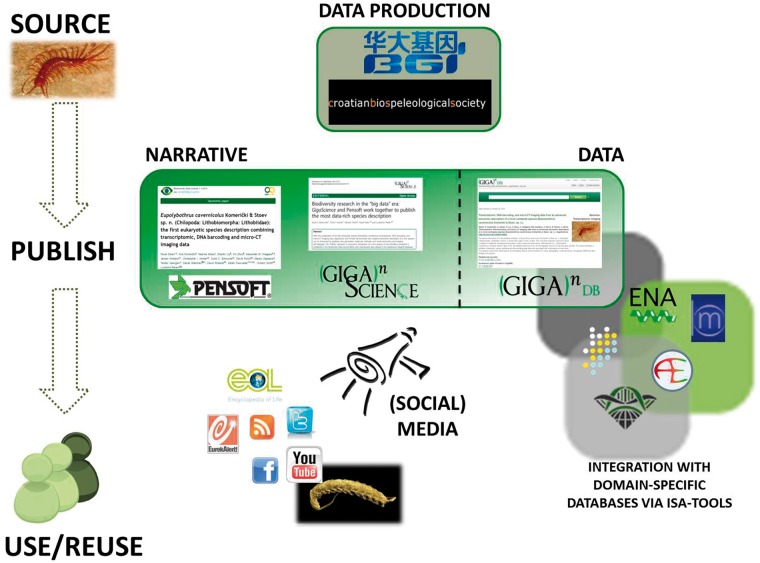
The integrated approach to data dissemination and attribution, using the example of the cyber-centipede data-rich species description.

A good example of the utility of GigaDB can be seen from recent collaboration between *GigaScience*, *China National GeneBank*, *BGI-Shenzhen* and *Pensoft Publishers*, which resulted in the publication of the first eukaryotic species description combining transcriptomic, DNA barcoding, morphology and X-ray microtomography imaging data. This ‘holistic’ approach in taxonomic description of a new species of cave-dwelling centipede was published in the Biodiversity Data Journal (24), with coordinated data release in GigaDB (25). It demonstrates how one can use the Internet to move accessibility of typed species out of museum collections and species description out of the print era. This attempt at a data-rich ‘cybertype’ mixed traditional morphological description, a transcriptomic profile, a 3D X-ray view and a movie of the living specimen to document important traits of its behavior. Figure 2 shows the extent and variety of data and metadata associated with this publication, all of which are collected together in, or linked to from, GigaDB. It also illustrates how multiple different media can be successfully integrated using the power of the ISA-tab metadata format. This study demonstrates how classical taxonomic description of a new species can be enhanced by applying new-generation molecular methods and novel computing and imaging technologies.

## Searching for data sets

The recently released new GigaDB Web site includes an improved full-text search facility powered by the open-source software package, Sphinx (http://sphinxsearch.com/docs/current.html), which allows users to easily find data sets. Registered users can also save searches and get automatic e-mail notifications about newly released data sets matching their criteria.

In addition to the keyword search, one can further filter the results on a number of fields to narrow the results to those most relevant. For example, if a search is performed for the keyword ‘cancer’, six data sets are currently returned containing 7852 files. These data sets can be further filtered to find human cancer data sets by using the common name ‘human’ to reduce the returned results to two data sets containing 5000 files. If registered and logged-in, users can save the search and filter criteria to their own GigaDB user area and be automatically notified of any new data sets matching those criteria.

## Future directions

GigaDB is currently operational and attracting an increasing number of users, but we are keen to ensure that we increase our usefulness to the research community by the addition of multiple new features over the coming months. Here, we highlight some of those features.

Although we try to host the materials and methods used in the data analyses, which are reported in *GigaScience* papers in GigaDB, it is often the case that this does not provide sufficient information to fully understand how the results of a scientific study were produced. A more comprehensive solution is required for our users to reproduce and reuse the computational procedures described in *GigaScience*. To this end, we are currently developing a data reproducibility platform, whereby data analyses are provided or re-implemented as Galaxy workflows (26). The first examples of this have already been published with testing of the *de novo* genome assembly tool, SOAPdenovo2 (27), and a population genomics toolkit (28). In the future, we hope to enable this Galaxy platform to directly access data stored in GigaDB through its API, which is currently in development. This API will also allow other computational tools to directly access the information held in our database.

In addition to the currently available methods of submission, we are developing an online wizard to allow our users an interactive online-guided submission process, which we hope will further encourage deposition of all available metadata by reducing the burden and suggesting the inclusion of data that may not have been considered relevant by the submitter.

Also in development is the implementation of a citation tracker to enable us to supply direct links to citations of GigaDB data sets, which will allow authors to see who, where and when their data sets are being reused. On top of the statistics that DataCite provides, we currently use Google Analytics internally to monitor traffic on the GigaDB Web site, which we hope will enable us to tailor future developments to areas of the Web site that lose traffic, but these data could also provide alternative statistics about the popularity of data sets.

GigaDB is supporting the ORCID registry (http://orcid.org/), and, where possible, we collect the ORCID identifier of any author(s) of data sets, allowing the linking of data sets by author to each other and to any external resources linked to those authors by ORCID identifiers. We hope to be able to extend the use of ORCID to allow users to log into GigaDB using their ORCID details. On top of external funding, we have received to set up this platform, some of the data hosting costs will have to be worked into the article processing charges of the journal with a view to our long term sustainability. With our low overheads, this will be a one-off payment that should be less than the fees and subscriptions that cloud storage and many other data publishing platforms and journals charge.

## Conclusions

Maximizing the reuse of published data does not only involve its deposition, along with its metadata, into an open-access repository in a standardized format. Results published in scientific articles also have to be reproducible so, for example, comparisons can be made with analyses on new research data. GigaDB is positioning itself to service the research community in these respects with the provision of its open-access database with citable DOIs.

Since its launch in 2011, GigaDB has been providing a platform for data dissemination and publication, but we wish to solicit open discussions from others, to ensure that future developments are focusing on the issues that need resolving. We are always keen to listen to our users and the scientific community to enable us to provide the tools and services that are required by researchers (database@gigasciencejournal.com).
